# RNA-seq analyses on gametogenic tissues of alfalfa (*Medicago sativa*) revealed plant reproduction- and ploidy-related genes

**DOI:** 10.1186/s12870-024-05542-2

**Published:** 2024-09-03

**Authors:** Fabio Palumbo, Giovanni Gabelli, Elisa Pasquali, Alessandro Vannozzi, Silvia Farinati, Samela Draga, Samathmika Ravi, Maria Cristina Della Lucia, Giovanni Bertoldo, Gianni Barcaccia

**Affiliations:** Department of Agronomy, Food, Natural resources, Animals and Environment, University of Padova, Legnaro, PD 35020 Italy

**Keywords:** *Medicago sativa* complex, WGCNA, Tau analysis, DEGs, Ploidy, Microsporogenesis

## Abstract

**Background:**

In alfalfa (*Medicago sativa*), the coexistence of interfertile subspecies (i.e. *sativa*, *falcata* and *coerulea*) characterized by different ploidy levels (diploidy and tetraploidy) and the occurrence of meiotic mutants capable of producing unreduced (2*n*) gametes, have been efficiently combined for the establishment of new polyploids. The wealth of agronomic data concerning forage quality and yield provides a thorough insight into the practical benefits of polyploidization. However, many of the underlying molecular mechanisms regarding gene expression and regulation remained completely unexplored. In this study, we aimed to address this gap by examining the transcriptome profiles of leaves and reproductive tissues, corresponding to anthers and pistils, sampled at different time points from diploid and tetraploid *Medicago sativa* individuals belonging to progenies produced by bilateral sexual polyploidization (dBSP and tBSP, respectively) and tetraploid individuals stemmed from unilateral sexual polyploidization (tUSP).

**Results:**

Considering the crucial role played by anthers and pistils in the reduced and unreduced gametes formation, we firstly analyzed the transcriptional profiles of the reproductive tissues at different stages, regardless of the ploidy level and the origin of the samples. By using and combining three different analytical methodologies, namely weighted-gene co-expression network analysis (WGCNA), tau (τ) analysis, and differentially expressed genes (DEGs) analysis, we identified a robust set of genes and transcription factors potentially involved in both male sporogenesis and gametogenesis processes, particularly in crossing-over, callose synthesis, and exine formation. Subsequently, we assessed at the same floral stage, the differences attributable to the ploidy level (tBSP vs. dBSP) or the origin (tBSP vs. tUSP) of the samples, leading to the identification of ploidy and parent-specific genes. In this way, we identified, for example, genes that are specifically upregulated and downregulated in flower buds in the comparison between tBSP and dBSP, which could explain the reduced fertility of the former compared to the latter materials.

**Conclusions:**

While this study primarily functions as an extensive investigation at the transcriptomic level, the data provided could represent not only a valuable original asset for the scientific community but also a fully exploitable genomic resource for functional analyses in alfalfa.

**Supplementary Information:**

The online version contains supplementary material available at 10.1186/s12870-024-05542-2.

## Introduction

According to Mendiburu and Peloquin [1], sexual polyploidization is a process leading to the formation of euploid zygotes due to the fertilization of gametes with a somatic number of chromosomes ≥ 2n. Unlike somatic and zygotic chromosome doubling [2], unreduced egg cells and/or pollen grains position sexual polyploidization as the prime driver in the origin and the evolution of polyploid plant species [3, 4]. This crucial mechanism underpins the cultivation of alfalfa, in particular, of the so-called *Medicago sativa* subsp. *sativa*–*coerulea*–*falcata* complex. This latter includes three main allogamous interfertile subspecies, either diploids (2*n* = 2*x* = 16) like *coerulea* and some *falcata* accessions or tetraploids (2*n* = 4*x* = 32) like *sativa* and some other *falcata* accessions [5]. The availability of interfertile subspecies with different ploidy levels, combined with the spontaneous formation of unreduced gametes boosts the constitution of sexual polyploids. In turn, this dynamic enhances the flow of genetic resources and supports cultivar improvement of *M. sativa*. Numerous studies emphasized the importance of ≥ 2n gametes in both the evolution [4, 5] and the breeding [6, 7] of alfalfa. This latter aspect is by no means irrelevant considering that alfalfa represents one of the most economically valuable crops in the world, with estimated annual sales higher than 10.8 billion dollars only in the USA [8]. Specifically, the occurrence of unreduced gametes represents a valuable resource for facilitating gene transfer from wild 2n accessions to cultivated 4n lines, through the fulfillment of breeding programs. Notably, some diploid mutant plants capable of producing unreduced gametes (2n) at high frequencies have been widely utilized in combination with tetraploids (naturally producing 2n gametes). This led to unilateral (i.e. unreduced gametes deriving from one of the two parents) sexual polyploidization (USP) events in the three main subspecies of the *Medicago* complex [6, 7, 9]. On the other side, bilateral (i.e. unreduced gametes deriving from both the two parents) sexual polyploidization (BSP) events have been experimentally accomplished by crossing two diploid mutant plants, both producing unreduced gametes (2n) [10, 11]. In both types of polyploidization schemes, a powerful triploid block was observed, giving rise to 100% tetraploid offspring in case of USP, and to both diploid and tetraploid progenies in case of BSP. This was probably due to the abortion of nearly all triploid embryos. This premature elimination may be triggered by abnormal endosperm development as a result of the distorted 2:1 ratio between the maternal and paternal genomes [12].

The significance of polyploidy remains a topic of debate. Some researchers judge the occurrence of polyploids as a mere and inconsequential result of a “rare mitotic or meiotic catastrophe” [13] traditionally leading to evolutionary “dead ends” [14]. However, recent genomic-based findings highlighted the crucial role of polyploidy in hybridization and speciation [15–17]. Moreover, from a breeding point of view, polyploids often exhibit three main advantages in comparison to their diploid counterparts, namely asexual reproduction, heterosis, and gene redundancy. The effect of polyploidization in autopolyploid species has been poorly investigated [18–20] and, despite the majority of natural polyploids are produced sexually thanks to the formation of unreduced gametes, the few studies conducted so far are mainly focused on polyploids obtained via somatic doubling. In forage crops, increased biomass and leaf size have been reported in naturally occurring tetraploid white clover (*Trifolium repens* L.), and red clover (*Trifolium pratense* L.) as compared to their diploid wild ancestors [21, 22]. In *Medicago* species, improved features such as leaf and stomatal size, epidermal cell surface, and increased green and dry biomass, were observed in tetraploid hybrids obtained through sexual polyploidization [11]. Innes et al., recently documented significant improvements in the cultivation of alfalfa tetraploids compared to their diploid counterpart, particularly regarding leaf area (+ 127%), autumn biomass (+ 106%), seed size (+ 58%), and canopy cover (+ 30%) [23].

The abundance of agronomical and phenotypical data relating to forage quality and production offers a deep understanding into the practical advantages of polyploidization, but it leaves unclarified most of the upstream molecular mechanisms in terms of gene expression and regulation. In fact, except for Rosellini et al. [11], who provided the first transcriptomic investigation on leaves of *M. sativa* synthetic polyploids, transcriptional data regarding floral reproductive whorls are lacking. Here, we tried to fill this gap by analyzing the transcriptome profiles of floral reproductive tissues of *Medicago sativa* synthetic polyploids flowers at four different stages (flower bud, closed flower, fully open flower and pollinated flower). Once verified the ploidy level of all the plants under examination through flow cytometry, RNA-seq analyses were performed to compare the expression profiles of diploid and tetraploid progenies obtained by BSP schemes (dBSP and tBSP, respectively) and tetraploid plants stemmed from USP schemes (tUSP). Weighted-gene network coexpression analysis (WGCNA), differentially expressed genes (DEGs) analysis, and tau analysis (τ) were initially run to detect time point-specific hub genes and molecular networks that distinctly describe each reproductive tissue, regardless of polyploidization scheme or ploidy level. Furthermore, polyploidization-sensitive differentially expressed genes (DEGs, from the comparison between dBSP vs. tBSP individuals) and species-sensitive DEGs (from the comparison between tBSP and tUSP individuals) were identified and discussed.

## Materials and methods

### Plant material and ploidy determination

**Fig. 1:**
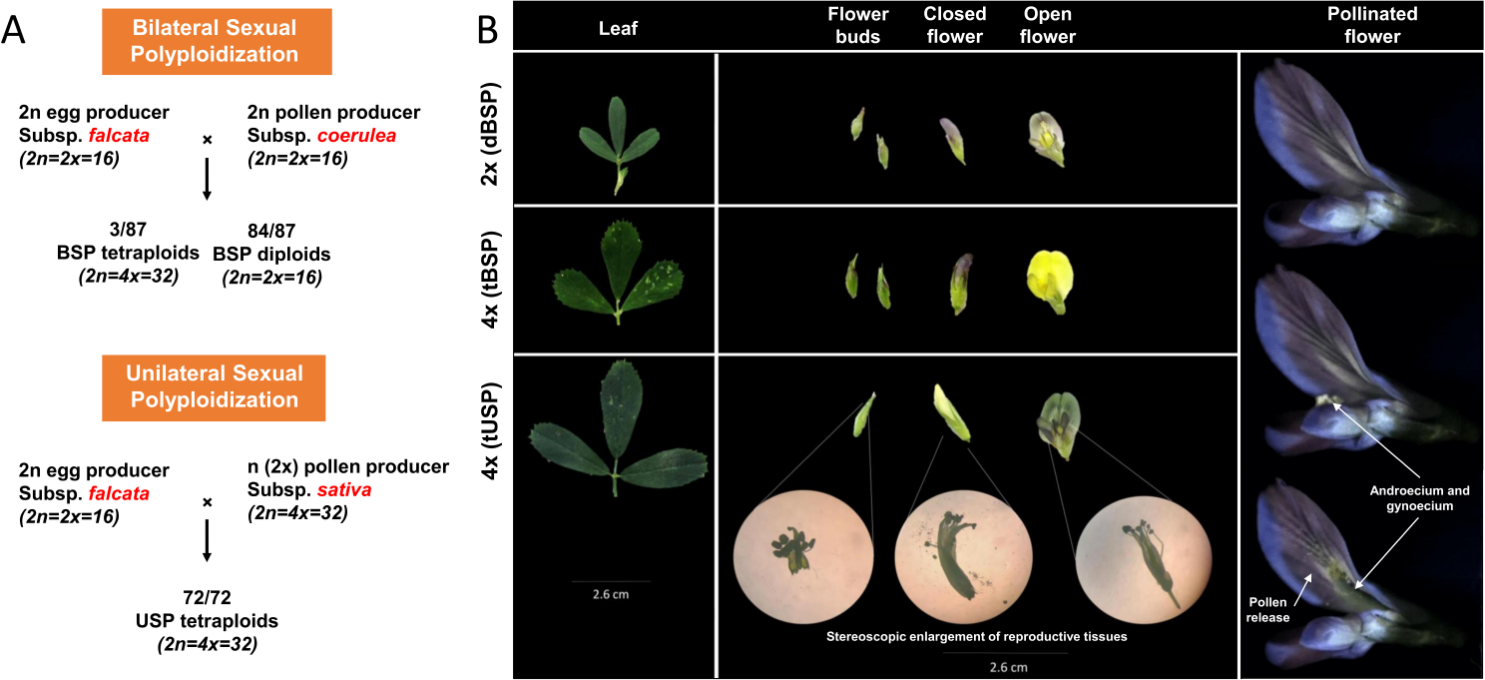
(**A**) Origins of the progeny analyzed in this study. BSP-deriving individuals (both diploids and tetraploids) derived from the *M. sativa* subsp. *falcata* mutant PG-F9 (2n = 2x = 16) able to produce 2n eggs [23, 24] × the 2n pollen producer *M. sativa* subsp. *coerulea* genotype 12-P (2n = 2x = 16) [25]. USP-deriving seeds were produced crossing the same 2n eggs producer used in the previous crossing (PG-F9 mutant) with a naturally tetraploid *M. sativa* subsp. *sativa* (variety Classe, 2n = 4x = 32) as pollen (*n* = 2x) donor. (**B**) Tissues sampled for each type of ploidy (i.e., dBSP, tBSP, and tUSP). For each biological replicate, leaf and flower tissues (anther and pistils) were collected, the latter at 4 different developmental stages: flower bud, closed flower, open flower, and pollinated flower

This story begins with some old seeds, produced around 25 years ago from two different crossing designs, namely BSP and USP, and forgotten at the bottom of a drawer. Individuals of the BSP progenies derived from the *M. sativa* subsp. *falcata* meiotic mutant PG-F9 (2n = 2x = 16) cytologically and genetically characterized as 2n egg producer [24–26] × the 2n pollen producer of *M. sativa* subsp. *coerulea* encoded as plant genotype 12-P (2n = 2x = 16) [27, 28]. These two 2n gamete producers were previously used for developing new synthetic tetraploids in alfalfa by sexual polyploidization programs [29] and for mapping unreduced spore and gamete production in diploid alfalfa using molecular markers [26, 28]. USP seeds were produced crossing the same diploid 2n eggs producer (PG-F9 mutant) with a naturally tetraploid *M. sativa* subsp. *sativa* (variety Classe, 2n = 4x = 32) as pollen (*n* = 2*x*) donor (Fig. 1A). Unfortunately, all three parents have been lost.

For each crossing, a hundred seeds were sown in growing boards and with a common potting soil at the “L. Toniolo” experimental farm of the University of Padova (Legnaro, Padova, Italy). After 4 weeks, 87 and 72 seedlings for BSP and USP matings, respectively, were transferred in two spaced plots.

For the screening of ploidy level, young leaves were sampled from each seedling and analyzed by flow cytometry. Briefly, 0.5 cm^2^ of leaves were chopped in a petri dish with sharp blades and 0.5 mL CyStain UV Precise P Nuclei extraction buffer (Sysmex Partec, Münster, Germany) were added to extract nuclei. After 5 min at room temperature, samples were filtered through a Partec 50 μm Cell Trics disposable filter (Sysmex Partec). Subsequently, the nuclei suspension was stained with 2 mL of 4′-6-diamidino-2-phenylindole (DAPI) staining solution (Sysmex Partec). After 5 min in the dark, samples were loaded in a CyFlow Cube 6 Ploidy analyzer (Sysmex Partec), detecting the relative fluorescence of total DNA of single nuclei. In each sample, the DNA content of at least 1000 nuclei was checked. Leaf samples of a wild alfalfa (4x) were used as external standards, alone and co-chopped with each sample.

### Samples collection

After ploidy determination, three samples (i.e. three biological replicates) were chosen among diploid plants deriving from BSP, three among tetraploid plants deriving from BSP and three among tetraploid plants deriving from USP. For each of the nine samples, we collected fully developed leaves (used as a control) and flowers at four different stages, namely flower buds, closed flowers (i.e. when colorful petals begin to appear) fully open flowers and pollinated flowers (i.e. 24 h after manually induced self-pollination). From each stage flowers, due to the small size of the reproductive system in alfalfa, we were forced to sample both anther and pistils together by removing the remaining part of the flower (i.e. sepals and petals, Fig. 1B). All samples were rapidly dissected in cold conditions, snap-frozen in liquid nitrogen and stored at -80 °C.

### RNA purification, library preparation, sequencing

For each sample, approximately 50 mg of frozen tissue were homogenized in a TissueLyser (Qiagen, Hilden, Germany) and total RNA was isolated using the “Spectrum Plant Total RNA Kit” (Sigma-Aldrich, St. Louis, MO, USA) according to the protocol provided by the company. The concentration was evaluated using both a Qubit 4 fluorometer with Qubit^®^ RNA BR Assay Kit (Thermo Fischer Scientific, Waltham, MA, USA) and a NanoDrop-1000 spectrophotometer (Thermo Scientific), thereby checking the quality in terms of 260/280 and 260/280 ratios. Finally, the integrity of the extracted total RNA was verified with a High Sensitivity RNA ScreenTape reagents on the 4150 TapeStation system (Agilent Technologies, MA, USA). Messenger RNA (mRNA) was isolated using oligo-dT beads (Dynabeads mRNA Direct Micro Kit, Thermo Fisher Scientific) following the protocol for mRNA isolation from purified total RNA. The quality and quantity were verified by TapeStation (Agilent Technologies), for detecting possible contaminations from 18 S to 28 S sequences. Sequencing libraries were prepared from mRNA samples using Ion Total RNA-seq Kit v2 (Thermo Fisher Scientific), following the instruction provided by the manufacturer, which include three main phases of mRNA fragmentation with Rnase III and purification, the hybridization and ligation of RNA, the reverse transcription and the final amplification of cDNA. The double-stranded barcoded cDNA libraries were eluted in 15 µl of nuclease-free water and their concentration and size distribution were assessed through D1000 screen Tape (Agilent TapeStation), then normalized to get a molar concentration of 100pM, and pooled. The sequencing run was performed using Ion 540 Chip on the Ion Torrent S5 System (Thermo Fisher Scientific).

### Raw sequencing data analysis

Quality control of the demultiplexed raw reads were assessed using MultiQC v1.13 [30]. Trimming of low-quality reads with a Phred score below 20 was done using cutadapt 1.9 [31]. The reads of all samples were aligned to the chromosome-scale assembly of *Medicago sativa* available in NCBI (PRJNA657344) [8]. The pipeline used bowtie2 v2.4.4 [32] at default parameters for read alignment, samtools v1.2 [33] for sorting and indexing the alignment files and bedtools multiCov v2.30.0 [34] for generation of counts matrix.

It should be noted that the *Medicago sativa* genome was not functionally annotated. Its annotation was therefore performed searching for orthologous proteins in the congeneric reference genome of *M. truncatula* (PRJNA431767) [35]. The protein sequences of *M. sativa* were used as query and aligned (BLASTp) against the barrel medic proteome-based database (Table S1).

### Weighted-gene correlation-network analysis (WGCNA)

Co-expression networks analyses were then conducted to pinpoint clusters (modules) of highly correlated genes associated to a given tissue/stage. For this type of analysis we opted for a DESeq2 normalization [36]. The counts matrix generated in Sect. 4, was firstly filtered by retaining only those genes having more than 5 aligning reads in at least three samples. Data were normalized using the median of ratios method with the R package DESeq2 [36], setting both the genotype (USP/BSP) and the tissue/stage of the samples as variables to be considered in the linear model. Using the same R package, a principal component analysis (PCA) was performed as well as a hierarchical clustering of the samples, in order to perform a first quality assessment of the analysis. The WGCNA was performed on the normalized data executing first a hierarchical clustering analysis with the *blockwiseModules* function from the R package *WGCNA* 1.70-3 [37]. The following parameters were used: net_type = signed, minModuleSize = 30, mergeCutHeight = 0.25, corType = Pearson, power = 8. The threshold parameter “power” was computed with the function *pickSoftThreshold* from the same package, selecting the value that determines the R^2^ value closest to 0.9.

The gene modules obtained were further corrected using a k-means clustering analysis with the *applyKMeans* function of the R package *CoExpNets* [38] and setting the following parameters: n.iterations = 50, net.type = signed, min.exchanged.genes = 20, excludeGrey = T. Those modules with the highest correlation to the tissues or stages being studied were further examined for gene significance (GS) and module membership (MM) using dedicated functions in *WGCNA* 1.70-3 package [37]. A GO enrichment analysis was run by means of the online tool ShinyGO 0.77 [39] for each of the modules identified.

### Differentially expressed gene (DEGs) determination

In parallel to the WGCNA analyses, DESeq2 normalized data were also used to carry out DEGs investigations with the R package DESeq2 [36]. Analyses were performed both to identify DEGs between different tissues/stages (regardless ploidy level or USP/BSP origin) and, at the same time, to detect, within each specific tissue/stage, DEGs capable of differentiating contrasting patterns of ploidy (i.e. dBSP vs. tBSP) or crossing (i.e. tBSP vs. tUSP). Genes were considered as DEGs if the adjusted p-value (padj) was lower than 0.05. The p-values were adjusted with the Benjamini-Hochberg method [40].

### Identification of tissue/stage-specific genes by means of tau analysis

The procedure elaborated by Kryuchkova-Mostacci and Robinson-Rechavi was adopted to calculate the τ (tau) indicator as an expression of the tissue-specificity level of each gene. This index ranges from 0 (for widely expressed genes) to 1 (for tissue/stage-specific genes) [41].

Starting from the raw count matrix produced in Sect. 4, a Transcript Per million (TPM) normalization was performed using *bioinfokit* v2.0.3 [42]. For each of the five tissues (i.e. leaf and reproductive tissues at four different floral developmental stages), the nine samples (three dBSP, three tBSP and three tUSP) were all considered as biological replicates, regardless the ploidy level or the type of crossing. The mean TPM value of a gene in a specific tissue ($\:\overline{\text{T}\text{P}\text{M}}$) was calculated as the arithmetic mean of the TPM values of the gene in the 9 biological replicates within the same tissue. Each gene having $\:\sum\:_{\text{i}=1}^{N}\overline{{\text{T}\text{P}\text{M}}_{\text{i}}}$ < 9 (where *N* is the total number of tissues) was discarded. The *log2Tran* function, included in the R package *tispec* [43], was used to perform a logarithm transformation, setting to zero all the negative values. *QuantNorm* function (from the same R package abovementioned) was applied for a quantile normalization of the whole dataset and to assign each gene a BIN value varying from 0 (lowest expression) to 10 (highest expression). Finally, *calcTau* allowed to estimate the specificity of each gene, applying the *τ* algorithm:

$$\:\tau\:=\frac{\sum\:_{\text{i}=1}^{N}(1-{x}_{i})}{N-1}$$

where *N* represents the tissue number and $\:{x}_{i}\:$is the expression value normalized by the highest expression.

Genes expressed exclusively in a single tissue/stage (*τ* = 1) were defined as Absolutely Specific Genes (ASGs); on the other hand, those genes whose expression was relatively high in a given tissue/stage (*τ* value ≥ 0.85 but < 1) were considered as Highly Specific Genes (HSGs). In addition, for each tissue, τ expression fraction (*τ*_*ef*_) was computed for all genes as follows:

$$\:{\tau\:}_{ef}=\tau\:\frac{qn}{max}$$

where *qn* is the quantile normalized expression and *max* is the highest quantile normalized expression.

The *getTissue* function assigned each gene (in each tissue) a score value ($\:\tau\:$ score) between 0 and 2, resulting from the sum of the *τ*_*ef*_ value and the normalized expression value of each gene (ranging from 0 to 1).

## Results and discussion

### Both BSP and USP crossings lead to polyploidization events

Alfalfa seeds proved an exceptional germination rate (72% and 87% for USP and BSP populations, respectively), despite having remained on the bottom of a drawer for 25 years, without any conservation measure and protected only by a paper bag.

**Fig. 2:**
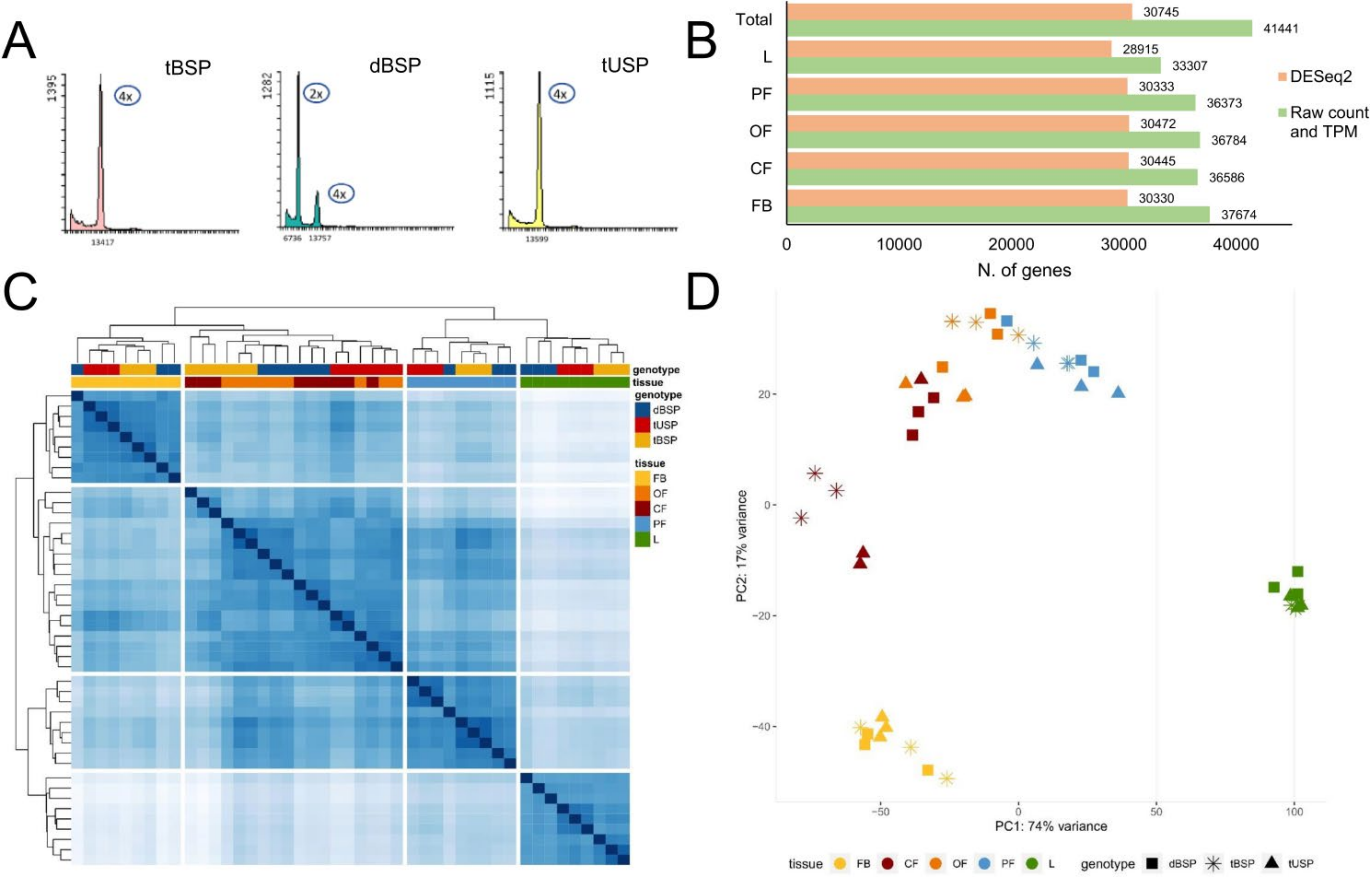
(**A**) Profiles derived from flow cytometry analyses conducted on tBSP, dBSP, and tUSP samples. (**B**) Total number of expressed genes for each of the four floral stages under study (flower bud FB, closed flower CF, open flower OF, and pollinated flower PF) and leaf (L), regardless the origin and the ploidy level. For each stage/tissue, transcripts were normalized using both TPM (transcripts per million) and DESeq2 approaches. (**C**) Cluster dendrogram analysis based on DESeq2 normalized data. (**D**) PCA analysis based on DESeq2 normalized data

From the flow cytometry analysis of the seedlings, all 72 USP samples were found to be tetraploids. Out of 87 individuals from the BSP crossing, three were tetraploids, whereas the remaining resulted diploids. Examples of the flow cytometry profiles obtained from the three types of plants under study (i.e. dBSP, tBSP and tUSP) are shown in Fig. 2A.

In both types of crossing, a strong triploid block prevented the development of triploid embryos, as already observed in several species such as *Dactylis glomerata* [44], *Achillea borealis* [45], *Brassica oleracea* [46] and *Medicago sativa* itself [6]. Furthermore, the percentage of tBSP (i.e. 3.5%, 3 out 87 samples) is in agreement with the experiment conducted in 1998 by Barcaccia et al., in which, from the crossing of the same parents (PG-F9 × 12-P), 5% of the resulting generation was tetraploid [10].

### RNA-seq analyses and data normalization

RNA-seq data were generated from fully developed leaf (L) and reproductive tissues (anthers and pistils), the latter at 4 stages of development (i.e. flower buds, FB; closed flowers CF; fully open flowers, OF; and pollinated flowers, PF). Tissues were isolated from 9 plants, namely three tBSP, three dBSP and three tUSP. The Ion Torrent S5 platform produced 514.8 M of reads (11.7 M on average per sample) and, after filtering and trimming step, 442.5 M of reads were retained for further analyses. From the raw counts matrix (Table S2) and, consequently, the TPM normalization (Table S3), 41,441 genes (out of the 47,202 available from the *Medicago sativa* genome assembly, PRJNA657344) resulted expressed in at least one tissue. L had the lowest number of expressed genes (33,307), whereas FB had the highest one (37,674, Fig. 2B). In addition to TPM normalization, the raw counts matrix was also used for DESeq2 normalization. From DESeq2 normalization, 30,745 genes resulted expressed in at least one tissue (Table S4). L showed again the lowest number of expressed genes (28,915), whereas OF had the highest one (30,472, Fig. 2B). The cluster dendrogram analysis and the PCA, both based on DESeq2 normalized data (Fig. 2C and D), showed a good correlation among the 9 samples of each tissue/stage, in particular for L and for reproductive tissues dissected from FB and PF. A slight overlapping was instead observed between the reproductive tissues sampled from CF and OF. In particular, considering the tUSP, one of the three CF biological replicates grouped with the three OF biological replicates (Fig. 2C and D). This could suggest a partial redundancy in the transcriptomic events occurring in these two chronologically contiguous floral stages.

Going deeper (i.e. within each tissue/stage), an excellent correlation was observed among the three biological replicates of each genotype. The only exception was represented by the three biological replicates of dBSP that, in two cases (FB and PF), did not perfectly cluster together (Fig. 2C).

To investigate genes involved in reproductive processes, we mainly focused on the FB stage, where the sporogenesis and gametogenesis processes take place [47]. All the data produced and analyzed were deposited in GeneBank (SRR28913430-SRR28913474) or provided as supplementary materials.

### Combining different approaches for the identification of flower bud specific genes

#### WGCNA and DEGs analyses

A WGCNA was carried out to identify genes with similar expression profiles related to leaf and to different floral developmental stages in alfalfa. This analysis was conducted regardless the ploidy (diploidy or tetraploidy) and the origin (BSP or USP) of the plants, by analyzing the 9 replicates of each stage/tissue together. This approach became one of the most successful methods for RNA-seq data in many plant species including, only in the last two years, orchid [48], Fig. [49], blueberry [50], grapevine [51] and tea plant [52]. To the best of our knowledge, this is the first time the WGCNA is applied to *M. sativa*.

**Fig. 3:**
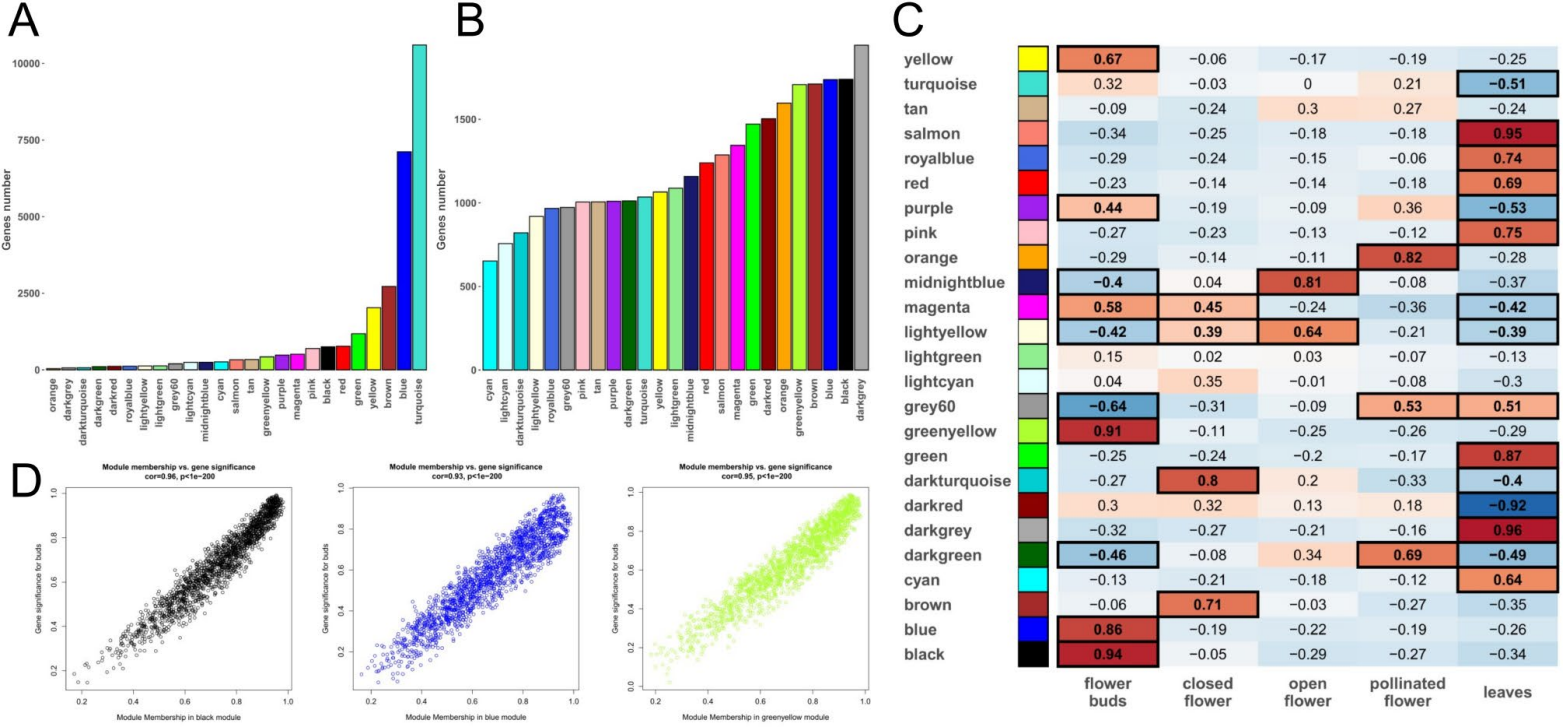
Weighted-gene correlation-network analysis (WGCNA) results. (**A** WG)CNA modules. (**B**) WGCNA modules after the K-means clustering analysis. (**C**) Correlation analysis performed between the 25 WGCNA modules obtained after the K-means clustering analysis and the 5 tissues under study to identify highly tissue-specific modules. The boxes with the thicker border represent modules with a correlation p-value < 0.01. (**D**) Tight correlation between gene significance (GS) and module membership (MM) in the three WGCNA modules that best correlate with FB tissues (i.e. black, blue, and greenyellow modules)

The 30,745 expressed genes resulting from DESeq2 normalization were firstly used to build a matrix raised to a soft-thresholding power β = 8 to ensure a scale-free network. By setting the minimum module size to 30 (a module is a cluster of highly correlated genes) we identified 64 distinct modules. In turn, all the modules with module eigengenes (ME, namely a value representative of the expression profiles of all the genes included in a module) correlating for values higher than 0.25 were merged, reducing the number of modules to 25, each marked with a specific color (Fig. 3A).

As highlighted by Botía et al. [38], the results of hierarchical clustering are highly dependent on the method used to calculate distances between clusters. Furthermore, once an expression vector is assigned to a cluster, it cannot be revised, even if the centroid of the module significantly diverges from it after subsequent additions of other expression vectors. On the other hand, a well-known flaw of k-means clustering is the need to set the number of clusters a priori [53]. This decision often relies on randomization methods (e.g. k-means++ [54]) or repeating the analysis with different parameters [55]. The method proposed by Botía et al. [38] corrects the flaws of both clustering algorithms. A first draft of the modules is done hierarchically. The subsequent k-means step starts from this draft, with the number of modules already set and the initial centroids determined, corresponding to the eigengenes of each module. The clusters are thus revised, and genes prematurely assigned to the wrong module are re-assigned.

Figure 3B shows the new distribution of the genes within the 25 modules after the K-means clustering analysis. The correlation analysis performed between the 25 newly organized modules and the 5 tissues under study allowed the identification of one or more highly correlating modules for each tissue (correlation p-value < 0.01, Fig. 3C). For example, the blue, greenyellow and black modules resulted specifically (*r* = 0.86, 0.91 and 0.94, respectively) and significantly (*p* = 4.4e-14, 2.2e-18 and 2.2e-22, respectively) correlated with FB tissues (Fig. 3C).

Within each module, we also investigated two additional parameters: (i) the module membership (MM), namely a value ranging from 0 to 1 describing the association between the profile of expression of a specific gene and the ME; and (ii) the gene significance (GS) which can take on positive or negative values, providing an estimate of the biological significance of a gene. Hypothetically, genes with both high GS and MM values are expected to play an important biological role within the tissue correlating with the module they belong to [37]. In Table S5 are reported the MM and GS of each gene assigned to a specific module in the WGCNA analysis. In Fig. 3D are reported three plots illustrating the correlation between GS and MM in the three modules (i.e. black, blue, and greenyellow) that best correlate with FB tissue. It seems evident how GS and MM are directly proportional. To determine whether tissues-associated modules exhibited an enrichment of genes within specific ontological categories, we performed a gene set enrichment analysis (GSEA) on genes meeting the criteria of MM > 0.9 which were identified as WGCNA hub genes.

**Fig. 4:**
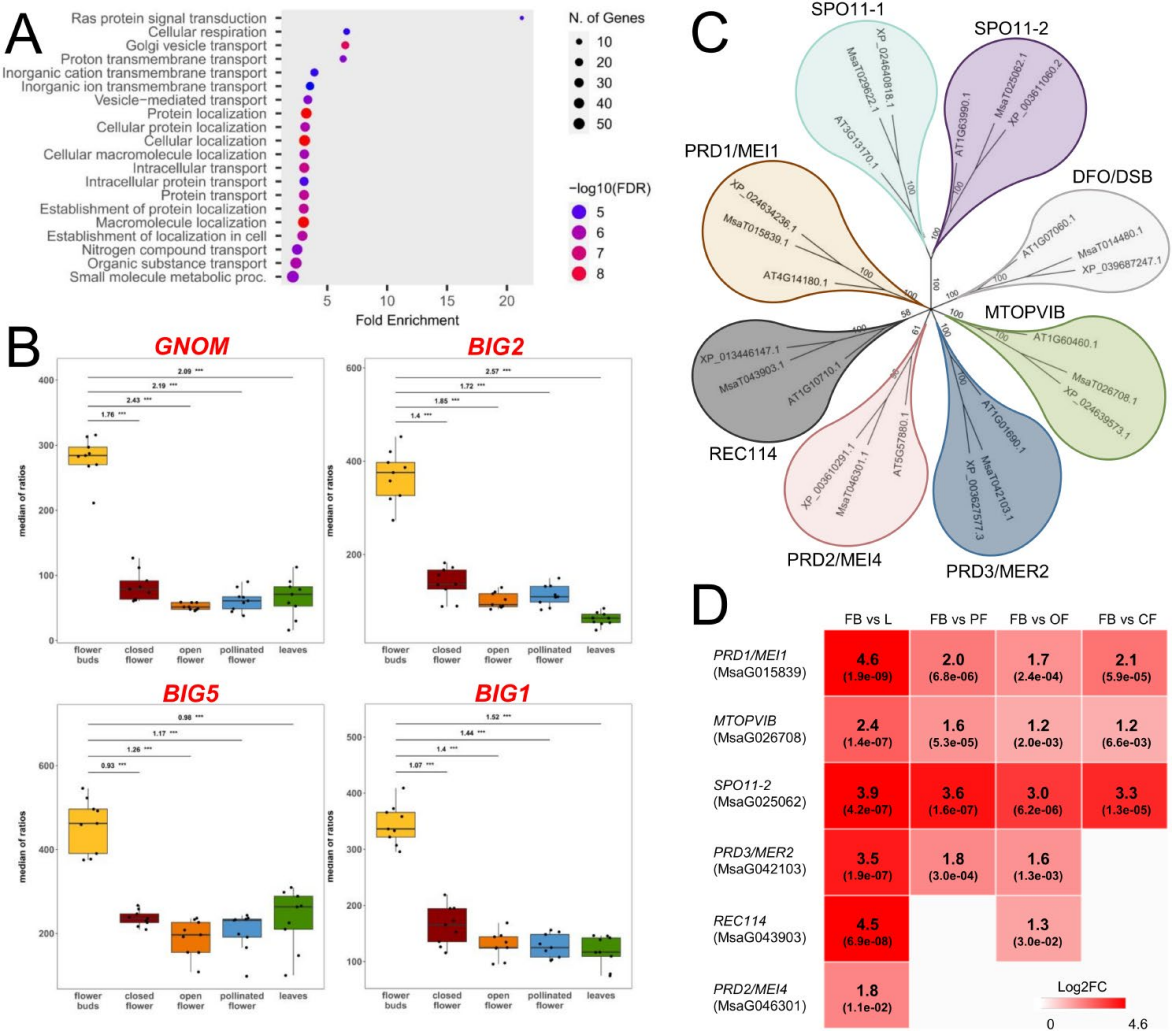
(**A**) GO enrichment analysis (Biological Process category) of the genes contained in the flower bud (FB)-associated black module. (**B**) DESeq2 normalized expression data for *BIG1*, *BIG2*, *BIG5* and *GNOM* genes, within the five tissues under study regardless the origin and the ploidy level. These genes were all included in the “Ras protein signal transduction” GO category (GO:0007265) and resulted all differentially expressed in FB in comparison to all the other tissues. (**C**) Dendrogram illustrating the phylogenetic relationships between the DSB machinery proteins of *A. thaliana* and their putative orthologues in *M. sativa* and *M. truncatula*. (**D**) Log2FC-based heatmap of six genes involved in the DSB machinery and significantly upregulated in FB in comparison to each other tissue. Only significant log2FC values (padj < 0.05) were reported (Padj is indicated in parentheses)

The most enriched Biological Process categories resulted “glutamate metabolic process” (GO:0006536) for the blue module, “cytoplasmic translational initiation” (GO:0002183) for the greenyellow module and “Ras protein signal transduction” (GO:0007265) for the black module. Noteworthy, this latter showed a fold enrichment of 21.2, with 6 genes (out of a total of 15 genes involved in this GO category) all located in the black module and with a GS > 0.80 (Fig. 4A).

Among these six genes, *BIG1*, *BIG2*, *BIG5* and *GNOM* stand out as members of the RAS GTPases superfamily and, in particular, of the ARF family, involved in vesicular trafficking [56]. In Arabidopsis, *BIG1* and *BIG2* are functionally redundant genes mediating secretion of the auxin efflux transporter PIN1, *BIG5* (also known as *BEN1*) is involved in the constitutive endocytosis of PIN1 whereas *GNOM* is known to mediate the polar recycling of the PIN1 from endosomes to the basal plasma membrane [57–59]. PIN1, in turn, is pivotal for female gametophyte development: when down-regulated or mutated, embryo sacs growth arrests at the mono- and/or bi-nuclear stages [60]. Based on the DESeq2 normalization, the above-mentioned genes showed an impressive transcript accumulation in FB in comparison to all the other tissues as reported in Table S4 and Fig. 4B. In particular, in the pairwise comparison between FB and each other tissue, these genes resulted always statistically significant DE (padj ≤ 0.001; ***; Table S6).

It should be noted that in the same WGCNA black module there were also three genes encoding for RAS-group Leucine-rich repeat (LRR) proteins (PIRLs): *PIRL1*, *PIRL6* and *PIRL9*. PIRL proteins are known to interact directly with the Ras GTPases superfamily, taking part in signal transduction. In Arabidopsis, *PIRL6* is a flower specific gene whose knockdown induces abnormal or aborted pollen formation and arrest in the embryo sac [61], whilst *PIRL1* and *PIRL9* act redundantly in male gametophyte formation, resulting pivotal for differentiation of microspores into pollen [62]. Also in this case, in the pairwise comparison between FB and each other tissue, these genes resulted always statistically significant DE (padj ≤ 0.001; ***; Table S6). Overall, the high expression levels found in FB for genes encoding for both Ras GTPases proteins (i.e., BIG1, BIG2, BIG5, and GNOM) and Ras GTPases-interacting proteins (i.e., PIRL1, PIRL6, and PIRL9), along with the involvement of their Arabidopsis orthologues in reproductive processes, led us to hypothesize that these genes could act similarly in alfalfa. However, thorough functional studies are required to determine the actual role of each of the aforementioned genes in the production of functional pollen grains and embryo sacs.

Compatibly with the sporogenesis events typical of the floral stage under consideration (FB), within the three modules associated with the FB (i.e. blue, black and greenyellow) we also identified some putative orthologues involved in genetic recombination and, in particular, in the DNA double-stand breaks (DSB) machinery. This latter is constituted by a Topo VI heterotetramer enzyme (in turn, composed of SPO11-1, SPO11-2 and two MTOPVIB subunits [63]) and some accessory proteins like PRD1 (also known as MEI1), PRD2 (MEI4), PRD3 (MER2), DSB (DFO) and PHS1 (REC114) [64]. The knockdown of most of these genes was found to lead to a disruption of male and female meiosis and to a lack of embryo sac and pollen development [65]. Based on BLASTP analyses we identified the putative orthologues of the aforementioned proteins also in *M. sativa* and *M. truncatula* and their phylogenetic relationship are illustrated in Fig. 4C. The putative orthologues of *SPO11*-2, *MTOPVIB* and *PRD1* in alfalfa resulted always significantly upregulated in the pairwise comparison between FB and each other tissue, whereas *PRD3/MER2*,* REC114* and PRD2/MEI4 resulted upregulated only in some of the comparisons (Fig. 4D, Table S6).

#### Tau (τ) analyses

The relative expression of a specific gene in one tissue compared to others can vary, and in certain situations, it is more valuable to pinpoint genes exclusively expressed in one tissue and not in others. To identify specific tissue gene markers, we employed the tau (τ) algorithm, commonly utilized in transcriptomic studies of animals or humans. This algorithm assesses the tissue-specificity level of a gene transcript in a specific genome.

**Fig. 5:**
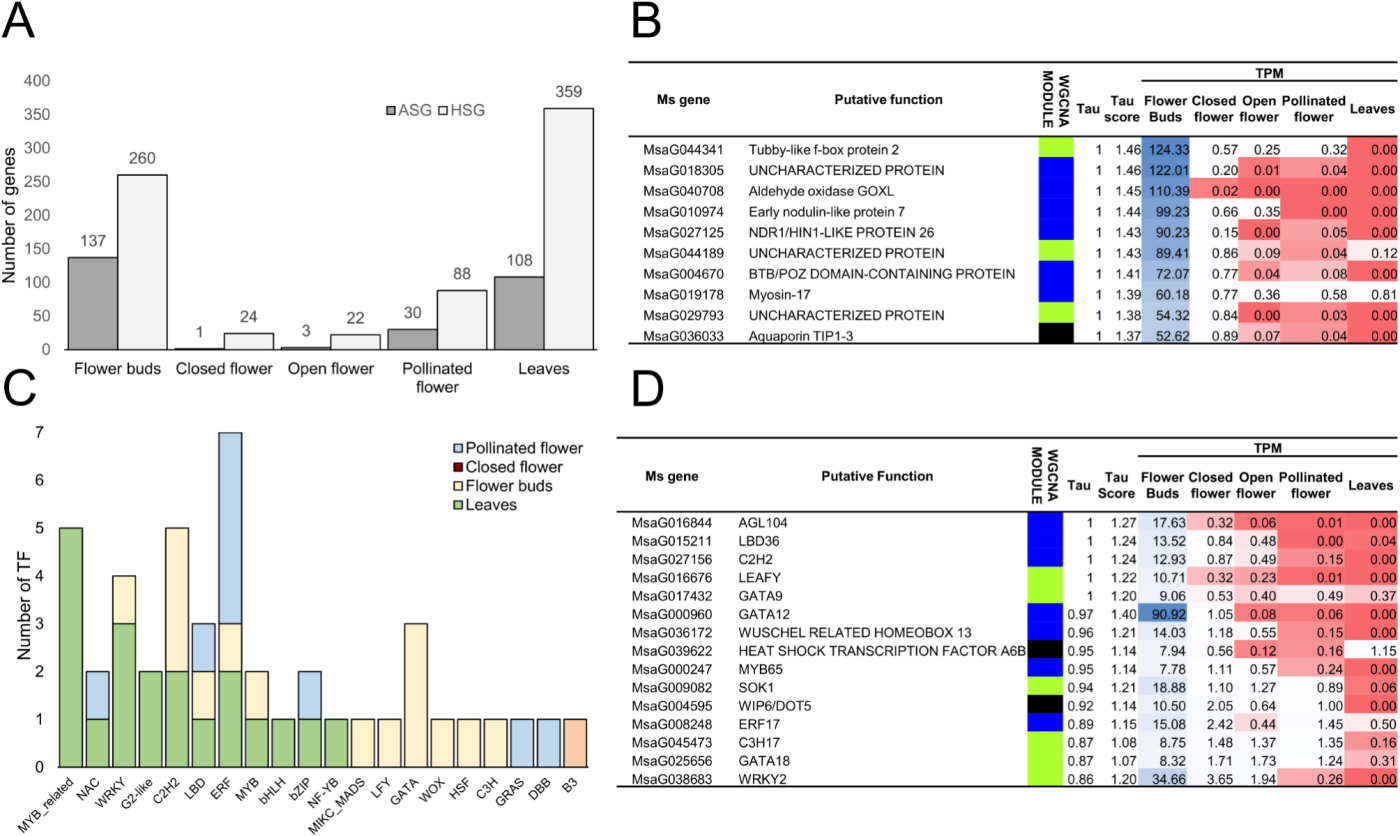
(**A**) Histograms displaying the distribution of Absolutely Specific Genes (ASG; tau = 1) and Highly Specific Genes (HSG; tau ≥ 0.85 and < 1) over the five tissues/stages considered regardless the origin and the ploidy level. (**B**) The top 10 ASG in FB ranked according to the tau score and TPM. For each gene the putative function, the belonging WGCNA module, tau, tau score, and TPM in each tissue/stage are reported. To facilitate understanding, a 2-colors scale (red to blue)-based conditional formatting was applied to the cells containing TPM values, where red indicates the lowest values whereas blue is used for the highest ones. (**C**) Highly Specific and Absolutely Specific Transcription Factors (respectively, HSTF and ASTF) specifically expressed in various tissues/stages analyzed in this study. D) HSTF and ASTF identified in FB and ranked according to the tau. For each gene the putative function, the belonging WGCNA module, tau, tau score, and TPM in each tissue/stage are reported. To facilitate understanding, a 2-colors scale (red to blue)-based conditional formatting was applied to the cells containing TPM values, where red indicates the lowest values whereas blue is used for the highest ones

After the quantile normalization of 18,364 genes (each gene having $\:\sum\:_{\text{i}=1}^{N}\overline{{\text{T}\text{P}\text{M}}_{\text{i}}}$ < 9, with *N* is the total number of tissues) and the creation of BIN profiles, the use of the τ algorithm allowed to assign a value between 0 (indicating constitutive expression in all or most tissues) and 1 (indicating absolute specificity for a particular tissue) to each gene. The distribution of τ values across the gene set is depicted in Fig. 5A and aligns with the expectations based on Kryuchkova-Mostacci and Robinson-Rechavi [41].

Out of the total, 750 genes demonstrated high specificity (HSG, 0.85 ≤ τ < 1), and 279 were found to be absolutely specific (ASG, τ = 1) (Table S7). As expected, leaf (L), being the only non-floral tissue, exhibited the highest number of highly specific genes (HSG, 359). The top 10 ASG genes of this tissue resulted mainly involved in basal abiotic and biotic stress response, such as *DRN1* (*DISEASE RELATED NONSPECIFIC LIPID TRANSFER PROTEIN 1* [66]), ATKTI5 (KUNITZ TRYPSIN INHIBITOR 5 [67]), and *CRK10* (*cysteine-rich RLK* [68]) or in photosynthetic processes, such as *RBCS1A* (RIBULOSE BISPHOSPHATE *CARBOXYLASE* SMALL CHAIN 1 A). Within the floral kinetics, FB had both the highest numbers of HSG (260) and ASG (137), in agreement with the specific sporogenesis and gametogenesis events occurring in this tissue (Fig. 5A).

Among the ASG of FB, in Fig. 5B we reported the top 10 genes, ranked according to the TPM values. According to the literature, four out of ten were found to be involved in the male and female gametogenesis process. The role of the Tubby-like F Protein 2 (TLP2) transcription factor (TF) in Arabidopsis is not fully elucidated but its transcripts resulted exceptionally abundant in pollen (its expression level is almost 10 times of other tissues) [69]. GOXL4, an aldehyde oxidase, seems to be specific to the locules of the developing anthers, most likely the tapetum. It is likely that GOXL4 is able to generate H_2_O_2_ being one of the contributors of the ROS-mediated programmed cell death of the tapetum [70]. TIP1-3, along with TIP5-1, is the only aquaporin gene that is selectively and highly expressed in pollen. In particular, TIP1-3 is specific for the vacuoles of the vegetative cell and tip1-3 mutants showed shorter than normal pollen tubes when germinated in vitro [71]. On the other hand, Early nodulin-like protein 7 was found to be specifically expressed within the female gametophyte [72, 73] although its role remains still unknown.

To identify transcriptional regulators specifically expressed in various tissues analyzed in this study, HSG and ASG were screened using functional annotations provided in the Plant Transcription Factor database (Plant TFDB) to identify Highly Specific and Absolutely Specific Transcription Factors (respectively, HSTF and ASTF). Leaf exhibited the highest number of HSTF [16] whereas, within the floral kinetics, FB had the highest numbers of ASTF [5] and HSTF (10, Fig. 5C and D). The putative Arabidopsis orthologous of some of the 15 ASTF/HSTF of FB were found to be involved in sporogenesis and gametogenesis processes. Among them we mention: AGL104, required for pollen maturation [74], LBD36 and GATA12 that were found to be specifically expressed in germ cell and pollen grains respectively [75, 76], MYB65 that participates (redundantly with MYB33) to the microsporogenesis process [77] and finally WRKY2 that, along with WRKY34 and VQ20, co-modulates multiple genes involved in pollen development [78].

It is interesting to note how both the top ten ASG (Fig. 5B) and the 15 HSTF/ASTF (Fig. 5D) identified in FB were all included in the three main modules of the WGCNA analysis that resulted specifically and significantly correlated with FB (black, blue and greenyellow, Fig. 3C), conferring greater robustness to the results obtained and paving the way for a subsequent phase focused on refining the selection of key genes of interest.

#### Comparison among WGCNA, DEGs and tau analyses and determination of key hub genes in flower buds

**Fig. 6:**
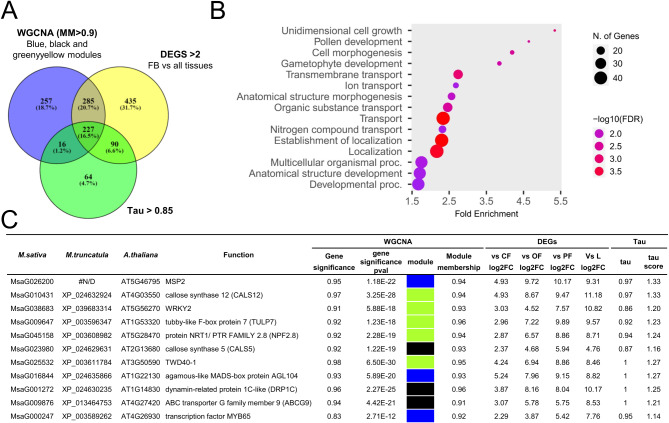
(**A**) Venn diagram showing specific and common genes among WGCNA (i.e. genes belonging to the three WGCNA modules with the strongest positive correlation with FB tissue, namely blue, black, and greenyellow and with module membership > 0.9), DEGs (genes differentially upregulated in FB compared to all other stages/tissues with log2FC > 2 and p-adj < 0.05) and tau (ASG and HSG genes in FB) analyses. The genes common to all three analyses (*n* = 227) were considered key hub genes of FB. (**B**) GO enrichment analysis (Biological Process category) of the Arabidopsis genes which were putative orthologous of the 227 key hub genes of *M. sativa.* (**C**) Detailed description of the 11 genes list involved in “Pollen development” GO category (GO:0009555) and belonging to the key hub genes of FB. For each gene the putative function, the putative orthologues in *M. truncatula* and *A. thaliana*, the belonging WGCNA module (with gene significance and module membership values), the log2FC values in comparison to all other tissues/stages, tau, and tau score, are reported

The comparison among the HSG and ASG identified in FB through the tau analysis (*n* = 397), the FB-specific modules pinpointed by WGCNA (blue, black and greenyellow module genes with MM > 0.9, *n* = 785) and the statistically significant DEGs with log2FC > 2 in any comparison between FB and each other tissue (*n* = 1,037), revealed a total of 227 genes shared among the three methodologies (Fig. 6A, Table S8).

The fact that the percentage of shared genes among the three analysis is only 16.5% is not unexpected, given that the modules isolated by WGCNA may encompass genes exhibiting elevated expression levels even in tissues not directly associated with the module itself, whereas HGS/ASG identified by the tau algorithm are those predominantly or even expressed in a particular tissue and not in others. Despite the distinct biological implications of the results yielded by the two analyses, we regarded the shared genes between the approaches as particularly noteworthy, designating them as key hub genes. Assessing genes based on both expression and specificity is valuable for researchers focusing on a single tissue, aiming to identify a set of genes highly specific to that tissue, abundantly expressed (facilitating laboratory bench work), and minimally expressed in other tissues (reducing off-target effects). Based on BLASTP and ShinyGO 0.77 [39], we retrieved and subjected to enrichment analyses the putative Arabidopsis orthologous of the 227 *M. sativa* key hub genes (Table S8). “Pollen development” (GO:0009555) was among the most enriched categories with 11 genes involved (Fold enrichment = 4.6, FDR = 3.0E-03) (Fig. 6B and C). Three of them, namely *WRKY2*, *AGL104* and *MYB65*, encodes for TFs already discussed in the previous section. Two callose synthases (CS), namely *CS5* (or glucane synthase 2, *GSL2*) and *CS12* (or *GSL5*) were also part of the “pollen development” category. In Arabidopsis there are at least 12 genes identified as callose synthases (or glucan synthase). Among them, *GSL2* and *GSL5* are involved in callose synthesis essential for pollen development, fertility, and/or viability [79]. In particular, *GSL5* have significant, albeit partially overlapping with *GSL1*, functions in both microspore and pollen grain development, contributing to the creation of the callose wall, which segregates the microspores within the tetrad, and to the pollen grain maturation [80]. On the other hand, *GSL2* was found to be required for exine formation in pollen wall during microgametogenesis [81].

Sporopollenin is enveloped by a mixture of compounds such as waxes, lipids, sterols, sugars, and proteins, collectively referred to as pollen coat or tryphine. This layer serves as the direct interface between the pollen and its external environment, and the two main aromatic constituents are represented by hydroxycinnamoyl spermidine conjugates (HCAAs) and flavonol-3-O-glycosides. One of the 11 hub genes identified in FB and involved in the “pollen development” process, namely *NPF2*.8 was reported to be essential for the accumulation and transport of pollen-specific flavonol glycosides to the pollen surface [82]. Along with the above-mentioned compounds, also steryl glycosides are critical for pollen fitness, by supporting pollen coat maturation. *ABCG9*, another pollen development-related gene identified through the integration of WGCNA, DEGs and tau analyses, resulted pivotal in the accumulation of this sterols on the surface of pollen [83]. Finally *DRP1C* is required for gametophyte development and, in particular, for plasma membrane morphology of the pollen grain and callose deposition [84].

In conclusion, although our discussion focused only on a small fraction of the 227 key hub genes identified in FB through the integration of three different approaches (WGCNA, tau, and DEGs), we believe that the gene list may represent a valuable tool for the scientific community. Specifically, we have identified a core of genes and transcription factors potentially involved in various stages of pollen production.

## Polyploidization-sensitive and species-sensitive differentially expressed genes

All the analyses (i.e. WGCNA, DEGs, and tau) conducted and discussed so far have allowed the identification of robust sets of genes involved in the development of floral tissues (four stages) and leaf tissues (one stage), regardless of the degree of ploidy (diploidy or tetraploidy) and the origin of the samples. For clarity, we remind that the results described above were obtained considering the 9 samples collected for each stage/tissue (three dBSP, three tBSP, and three tUSP) as biological replicates.

**Fig. 7:**
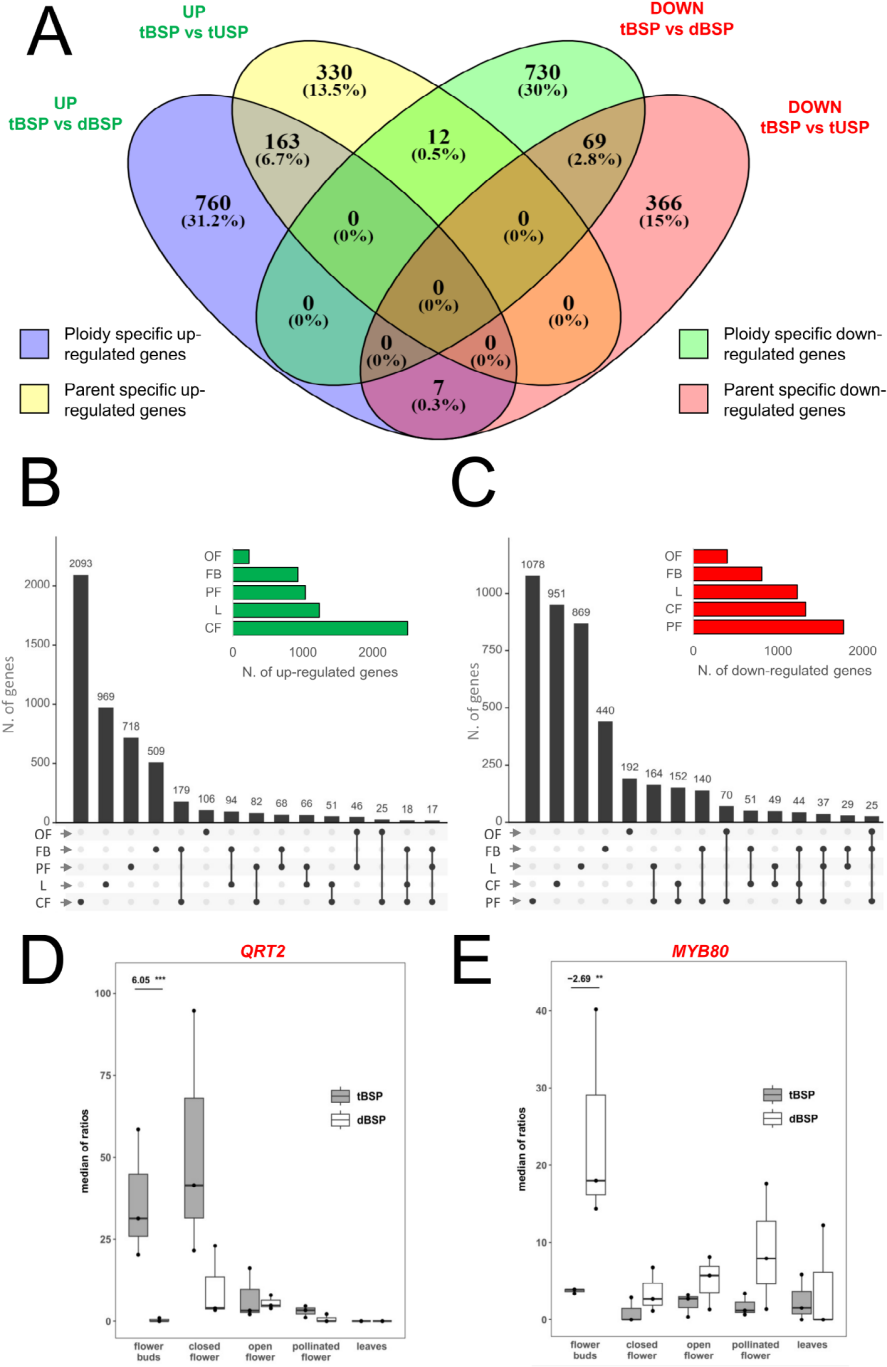
(**A**) Venn diagram showing specific and common DEGs calculated in flower bud (FB) between samples with different ploidies but derived from the same cross (tBSP vs. dBSP), and between samples sharing the same ploidy and the same female parent, but different male parents (tBSP vs. tUSP). (**B**) Upset plots showing the distribution across various tissues/stages of genes differentially upregulated in the comparison tBSP vs. dBSP. Single points indicate private upregulated genes identified in a given tissue/stage, whereas 2 to 5 dot plots indicate upregulated genes shared between 2 to 5 conditions. For example, in the comparison tBSP vs. dBSP, 509 genes resulted upregulated exclusively in FB. In the top right corner, the histograms show the total number of upregulated genes in each tissue/stage in the comparison tBSP vs. dBSP. (**C**) Upset plots showing the distribution across various tissues/stages of genes differentially downregulated in the comparison tBSP vs. dBSP. Single points indicate private downregulated genes identified in a given tissue/stage, whereas 2 to 5 dot plots indicate downregulated genes shared between 2 to 5 conditions. For example, in the comparison tBSP vs. dBSP, 440 genes resulted downregulated exclusively in FB. In the top right corner, the histograms show the total number of downregulated genes in each tissue/stage in the comparison tBSP vs. dBSP. (**D**) and (**E**) DESeq2 normalized expression data for *QRT2* and *MYB80* within the five tissues/stages under study in the comparison tBSP vs. dBSP

However, within the same tissue and developmental stage, we believe it could also be interesting to evaluate the changes that arise at the transcriptomic level, both considering samples with different ploidies but derived from the same cross (tBSP vs. dBSP), and samples sharing the same ploidy and the same female parent, but different male parents (tBSP vs. tUSP). The DEGs for tissue/stage between tBSP vs. dBSP, and between tBSP vs. tUSP, are reported in Tables S9 and S10, respectively. Within the same tissue/stage, intersecting the DEGs deriving from tBSP vs. dBSP and the DEGs deriving from tBSP vs. tUSP, it is possible to identify lists of genes that we may consider respectively “ploidy-dependent” or “male parent-dependent”. As an example, in Fig. 7A, the flower bud DEGs (i.e., upregulated and downregulated) identified in the two aforementioned comparisons are reported.

In FB, 930 genes were found to be up-regulated in the comparison tBSP vs dBSP, while 505 were up-regulated in the comparison tBSP vs tUSP. Among these, 163 (6.7%) were up-regulated in both comparisons, whereas 760 and 330 resulted specifically up-regulated in each comparison and could therefore be considered, respectively, ploidy-dependent and parent-dependent (Fig. 7A). An analogous discussion can be made for the down-regulated genes. Extending the same analysis to all the other tissues/stages (Fig. S1), we did not observe any specific trend in terms of ploidy or parent impact. Indeed, in FB, CF and L the number of upregulated and downregulated genes is consistently higher in the comparison between genotypes with different ploidies (i.e., tBSP vs dBSP) rather than between genotypes with different fathers (i.e., tBSP vs tUSP). Conversely, an opposite situation is observed in OP and PF, where the ‘parent effect’ seems to be more impactful than the ‘ploidy effect’.

On the contrary, the frequency of genes with contrasting patterns between the two comparisons was always extremely low in all the tissues/stages under study. For example, from Fig. 7A, it can be inferred that in FB only 12 genes are upregulated in the tBSP vs. tUSP comparison and simultaneously downregulated in the tBSP vs. dBSP comparison. Similarly, only 7 genes exhibited an opposite behavior, namely upregulated in the tBSP vs. dBSP comparison and simultaneously downregulated in the tBSP vs. tUSP comparison. These observations hold true extending the DEGs analysis in all the other tissues/stages (Fig. S1): the percentage of genes with contrasting patterns (i.e., up-regulated in one comparison and downregulated in the other) is consistently between 0 and 1.9% of the total genes.

In order to highlight ploidy-dependent genes, we decided to consider only the tBSP vs. dBSP comparison. In Fig. 7B and C we reported the distribution across all the tissues/stages of genes differentially upregulated or downregulated in the above-mentioned comparison. In two previous studies, it was demonstrated that the same tBSP plants, obtained by crossing inter-fertile subspecies (i.e., *M. sativa* subsp. *falcata* × *M. sativa* subsp. *coerulea*), are still capable of producing viable pollen, but its germinability is very low and a strong decline in fertility was observed [10, 85]. Specifically, the mean seed sets for diploid plants and tBSP group were respectively 0.115 and 0.006 in the case of self-pollination, and 1.873 and 0.031 in the case of cross-pollination [10].

To deepen some of the possible molecular mechanisms underlying the reduced fertility of tBSPs (in comparison to dBSP), we mainly focused on genes upregulated and downregulated exclusively in FB, namely the stage where the sporogenesis and gametogenesis processes are supposed to occur.

From Fig. 7B, it can be observed that 509 genes are upregulated exclusively in FB (Table S11). Based on the GO enrichment analysis, 26 of them were found to fall in the Biological Process category named “reproductive process” (GO:0022414). Among them, *QRT2* positioned in the top ten of the most upregulated genes (log2FC = 6.054, p-adj = 1.46E-04 Fig. 7D, Table S11). Earlier genetic studies have demonstrated that *QRT2*, along with *QRT1* and *QRT3*, is necessary during microsporogenesis for the separation of developing pollen grains. These three polygalacturonases are involved in pectin degradation, which, in turn, plays a pivotal role in cell adhesion. The lack of functionality of *QRT2* produces tetrad pollen in which microspores fail to separate [86]. On the contrary, an overexpression of *QRT2* causes male sterility. The authors explanation for this result is that increased *QRT2* expression leads to abnormal cell–cell adhesion, which inhibits gamete formation [86]. Also *CEP1* and *MIK2* fell in the “reproductive process” category and resulted strongly upregulated (log2FC = 5,100, p-adj = 1,15E-05 and log2FC = 4.400, p-adj = 4,50E-02, respectively Table S11) in FB, considering the tBSP vs. dBSP comparison. It was found that an overexpression of *CEP1* causes premature tapetal programmed cell death and pollen infertility [87] whereas high expression levels of MIK2 are associated with self-incompatibility reactions [88, 89].

*MYB80* (also known as *MS188* and originally, *MYB103* Fig. 7E) was instead among the 440 genes (Fig. 7C, Table S11) that resulted downregulated (log2FC = -2.69, p-adj = 2.6E-03) in FB, again in the frame of the tBSP vs. dBSP comparison. It was also among the 30 genes that were found to fall in the Biological Process category named “reproductive process” (GO:0022414). In Arabidopsis, mutations in *MYB80* are responsible for a male sterile phenotype. Its lack of functionality leads to a reduced expression of both *A6*, a potential β-1,3-glucanase implicated in callose dissolution, and *MS2*, a fatty acid reductase likely involved in sporopollenin synthesis and, consequently, in exine formation [90]. As a consequence, the tapetal cell wall is not degraded, the release of tetrads is markedly diminished and pollen grains appear to lack the exine. Identical findings were also reported in chicory [91] and rice [92].

Further studies are required to ascertain whether the simultaneous upregulation of *QRT2*,* CEP1* and *MIK2* and downregulation of *MYB80* observed in the comparison between tBSP and dBSP are indeed responsible for the significant reduction in fertility and seed set observed in the tetraploid plants.

Despite a reduced fertility and seed set, tBSPs are characterized by increased leaf size and biomass, compared to their diploid counterpart (dBSP). This emerged both in our study (Fig. 1B) and in the studies by Rosellini et al. [11] and Innes et al. [23]. For this reason, although the main focus of our research was the reproductive aspect, we thought it was worth examining the ploidy-dependent leaf genes in the tBSP vs. dBSP comparison (Table S12). Interestingly, according the GO enrichment analysis of the leaf genes upregulated in a ploidy-dependent manner, some of the most enriched Biological Process categories were “cell wall organization (GO:0071555)”, “external encapsulating structure organization (GO:0045229)”, and “anatomical structure morphogenesis (GO:0009653)” (Fig. S2). Being cell wall the main constituent of plant biomass, it is not surprising to observe in tetraploids an upregulation of genes involved in cell wall synthesis or in anatomical structure changes [93].

## Conclusion

In *Medicago sativa*, improved plant and crop traits associated to forage yield such as bigger leaf size and higher dry biomass have been demonstrated in tetraploid hybrids obtained from sexual polyploidization. The wealth of agronomic and phenotypic data related to forage quality and production provides valuable insights into the practical benefits of alfalfa polyploids. However, the transcriptional changes that arise in this species as a consequence of polyploidization events remain, to date, almost entirely unexplored. To take a first step in this direction, we analyzed leaves and reproductive tissues sampled at different stages of development from diploid and tetraploid accessions obtained both through BSP and USP. For each of the three types of ploidy, we generated 15 RNA-seq datasets for flower buds, closed flowers, open flowers, pollinated flowers and leaves (three replicates each). The investigation into the molecular mechanisms underlying the ontogenetic determination of pistils and anthers is indeed a subject of considerable interest mainly because reproductive tissues represent the two main actors involved in the production of unreduced gametes and thus polyploid individuals. Setting aside the different types of ploidy and considering the samples as a whole, we initially analyzed the RNA-seq data considering only the stage/tissue variable. WGCNA, DEGs and tau analyses were utilized and combined for investigating the transcriptomic data and pinpointing tissue-specific key hub genes. We mainly focused on the flower bud stage, the fulcrum of sporogenesis and gametogenesis processes.

Furthermore, we assessed the transcriptomic changes occurring within the same tissue and developmental stage in samples with contrasting ploidy but common origin (tBSP vs. dBSP) or with identical ploidy but different origin (tBSP vs. tUSP) to identify, respectively, ploidy-dependent and parent-dependent genes.

While this study primarily functions as a broad and robust investigation at the transcriptomic level, the extensive data provided could represent a valuable asset for the scientific community. We are confident that our data should prove valuable in the *M. sativa* complex where there is a lack of suitable and exploitable information on plant reproduction- and ploidy-related genes, and where considerable time and labor are involved in obtaining genotypes and populations amenable to genomic analysis.

### Electronic supplementary material

Below is the link to the electronic supplementary material.

Supplementary Material 1

Supplementary Material 2

Supplementary Material 3

Supplementary Material 4

Supplementary Material 5

Supplementary Material 6

Supplementary Material 7

Supplementary Material 8

Supplementary Material 9

Supplementary Material 10

Supplementary Material 11

Supplementary Material 12

Supplementary Material 13

Supplementary Material 14

## Data Availability

The datasets generated and analyzed during the current study are available in National Center for Biotechnology Information (NCBI) and can be accessed in the sequence read archive (SRA) database (https://www.ncbi.nlm.nih.gov/sra). The accession number is PRJNA1107370 and includes 45 accession items (SRR28913430-SRR28913474).
