# Supplementary figures and images for: RNA-seq analyses on gametogenic tissues of alfalfa (Medicago sativa) revealed plant reproduction- and ploidy-related genes
Source: BMC Plant Biol. 2024 Sep 3;24:826. doi: 10.1186/s12870-024-05542-2 (PMC11370029; doi:10.1186/s12870-024-05542-2)

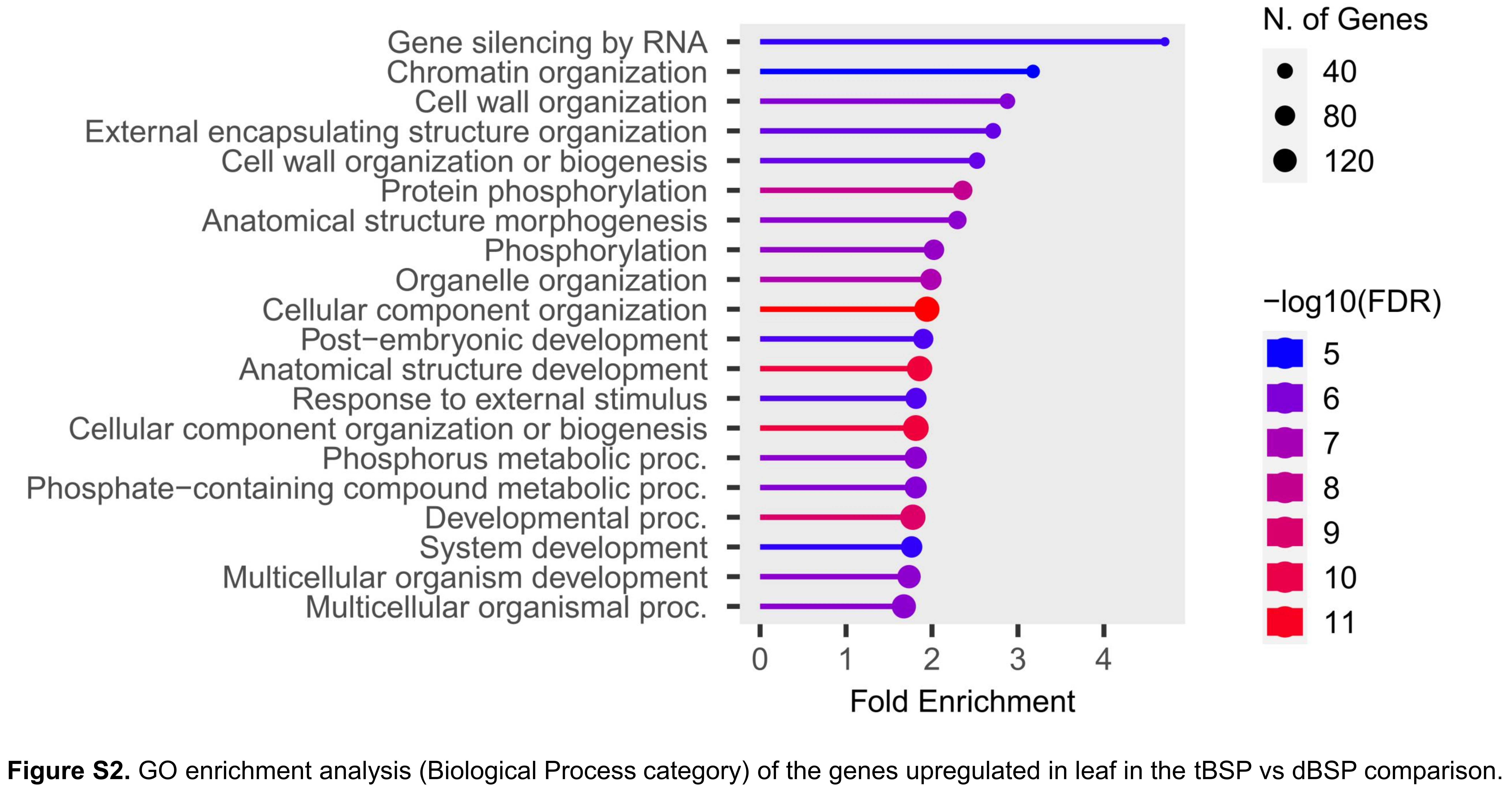

Supplement: Supplementary file 14 — Supplementary Material 14 [file 12870_2024_5542_MOESM14_ESM.jpeg]
